# Supplementary material for: A case for a negative-strand coding sequence in a group of positive-sense RNA viruses
Source: Virus Evol. 2020 Feb 10;6(1):veaa007. doi: 10.1093/ve/veaa007 (PMC7010960; doi:10.1093/ve/veaa007)
Supplement: veaa007_Supplementary_Data [file veaa007_supplementary_data.zip › Supplementary_figures_1b.pdf]

SUPPLEMENTARY MATERIAL

A case for a negative-strand coding sequence in a group of positive-sense RNA viruses

Adam M. Dinan, Nina I. Lukhovitskaya, Ingrida Olendraite and Andrew E. Firth

Division of Virology, Department of Pathology, University of Cambridge, Tennis Court Road, Cambridge, CB2 1QP, UK

Supplementary Table S1. Full list of sequences included in this study. Taxonomic information is taken from NCBI GenBank, where applicable.

**Organism**	**Database**	**Accession**	**Taxonomy of source**
Barns Ness breadcrumb sponge narna-like virus 4	GenBank	MF190030.1	Viruses, ssRNA viruses, ssRNA positive-strand viruses, no DNA stage, Narnaviridae
Beihai barnacle virus 10	GenBank	KX883482.1	Viruses, unclassified RNA viruses
Beihai narna-like virus 22	GenBank	NC_032404.1	Viruses, unclassified RNA viruses
Beihai narna-like virus 23	GenBank	NC_032457.1	Viruses, unclassified RNA viruses
Beihai narna-like virus 24	GenBank	KX883500.1	Viruses, unclassified RNA viruses
Beihai narna-like virus 25	GenBank	KX883471.1	Viruses, unclassified RNA viruses
Botrytis ourmia-like virus	GenBank	NC_028476.1	Viruses, ssRNA viruses, ssRNA positive-strand viruses, no DNA stage, Ourmiavirus
Cassava virus C	GenBank	NC_013111.1	Viruses, ssRNA viruses, ssRNA positive-strand viruses, no DNA stage, Ourmiavirus
Cryphonectria parasitica mitovirus 1-NB631	GenBank	NC_004046.1	Viruses, ssRNA viruses, ssRNA positive-strand viruses, no DNA stage, Narnaviridae
Epirus cherry virus	GenBank	NC_011065.1	Viruses, ssRNA viruses, ssRNA positive-strand viruses, no DNA stage, Ourmiavirus
Fusarium coeruleum mitovirus 1	GenBank	NC_026622.1	Viruses, ssRNA viruses, ssRNA positive-strand viruses, no DNA stage, Narnaviridae
Fusarium poae narnavirus 1	GenBank	LC150604.1	Viruses, ssRNA viruses, ssRNA positive-strand viruses, no DNA stage, Narnaviridae
Hubei mosquito virus 3	GenBank	NC_033257.1	Viruses, unclassified RNA viruses
Hubei narna-like virus 15	GenBank	KX883540.1	Viruses, unclassified RNA viruses
Hubei narna-like virus 16	GenBank	KX883526.1	Viruses, unclassified RNA viruses
Hubei narna-like virus 18	GenBank	KX883517.1	Viruses, unclassified RNA viruses
Hubei narna-like virus 19	GenBank	KX883542.1	Viruses, unclassified RNA viruses
Hubei narna-like virus 20	GenBank	KX883548.1	Viruses, unclassified RNA viruses
Hubei narna-like virus 21	GenBank	KX883539.1	Viruses, unclassified RNA viruses
Leptomonas Narna-like virus 1	GenBank	KY628364.1	Viruses, ssRNA viruses, ssRNA positive-strand viruses, no DNA stage, Narnaviridae
Leptomonas seymouri Narna-like virus 1	GenBank	KU935604.1	Viruses, ssRNA viruses, ssRNA positive-strand viruses, no DNA stage, Narnaviridae
Leptomonas seymouri RNA virus	GenBank	KX373304.1	Viruses, unclassified RNA viruses
Linepithema humile narna-like virus 1	GenBank	MH213236.1	Viruses, ssRNA viruses, ssRNA positive-strand viruses, no DNA stage, Narnaviridae
Narnaviridae environmental sample	GenBank	KP642119.1	Viruses, ssRNA viruses, ssRNA positive-strand viruses, no DNA stage, Narnaviridae
Narnaviridae environmental sample	GenBank	KP642120.1	Viruses, ssRNA viruses, ssRNA positive-strand viruses, no DNA stage, Narnaviridae
Ochlerotatus-associated narna-like virus 1	GenBank	KF298275.2	Viruses, environmental samples
Ochlerotatus-associated narna-like virus 2	GenBank	KF298276.2	Viruses, environmental samples
Ochlerotatus-associated narna-like virus 2	GenBank	KF298284.2	Viruses, environmental samples
Ophiostoma mitovirus 3a	GenBank	NC_004049.1	Viruses, ssRNA viruses, ssRNA positive-strand viruses, no DNA stage, Narnaviridae
Ophiostoma mitovirus 4	GenBank	NC_004052.1	Viruses, ssRNA viruses, ssRNA positive-strand

			viruses, no DNA stage, Narnaviridae
Ophiostoma mitovirus 6	GenBank	NC_004054.1	Viruses, ssRNA viruses, ssRNA positive-strand viruses, no DNA stage, Narnaviridae
Ourmia melon virus	GenBank	NC_011068.1	Viruses, ssRNA viruses, ssRNA positive-strand viruses, no DNA stage, Ourmiavirus
Phytophthora infestans RNA virus 4	GenBank	JN400241.1	Viruses, ssRNA viruses, ssRNA positive-strand viruses, no DNA stage, Narnaviridae
Phytophthora infestans RNA virus 4	GenBank	JN400242.1	Viruses, ssRNA viruses, ssRNA positive-strand viruses, no DNA stage, Narnaviridae
Phytophthora infestans RNA virus 4	GenBank	JN400243.1	Viruses, ssRNA viruses, ssRNA positive-strand viruses, no DNA stage, Narnaviridae
Phytophthora infestans RNA virus 4	GenBank	KU295722.1	Viruses, ssRNA viruses, ssRNA positive-strand viruses, no DNA stage, Narnaviridae
Phytophthora infestans RNA virus 4	GenBank	KU295726.1	Viruses, ssRNA viruses, ssRNA positive-strand viruses, no DNA stage, Narnaviridae
Phytophthora infestans RNA virus 4	GenBank	KU295727.1	Viruses, ssRNA viruses, ssRNA positive-strand viruses, no DNA stage, Narnaviridae
Point-Douro narna-like virus	GenBank	MF176258.1	Viruses, unclassified viruses
Saccharomyces 20S RNA narnavirus	GenBank	AF039063.1	Viruses, ssRNA viruses, ssRNA positive-strand viruses, no DNA stage, Narnaviridae
Saccharomyces 23S RNA narnavirus	GenBank	U90136.1	Viruses, ssRNA viruses, ssRNA positive-strand viruses, no DNA stage, Narnaviridae
Saccharomyces cerevisiae	GenBank	M64034.1	Eukaryota, Fungi, Dikarya, Ascomycota
Sanxia water strider virus 13	GenBank	KX883566.1	Viruses, unclassified RNA viruses
Shahe narna-like virus 4	GenBank	KX883556.1	Viruses, unclassified RNA viruses
Shahe narna-like virus 4	GenBank	KX883562.1	Viruses, unclassified RNA viruses
Soybean leaf-associated ourmiavirus 1	GenBank	KT598235.1	Viruses, ssRNA viruses, ssRNA positive-strand viruses, no DNA stage, Ourmiavirus
Tuber aestivum mitovirus	GenBank	NC_015629.1	Viruses, ssRNA viruses, ssRNA positive-strand viruses, no DNA stage, Narnaviridae
Wenling narna-like virus 7	GenBank	KX883602.1	Viruses, unclassified RNA viruses
Wenling narna-like virus 8	GenBank	KX883605.1	Viruses, unclassified RNA viruses
Wilkie narna-like virus 1	GenBank	NC_035126.1	Viruses, unclassified viruses
Wilkie narna-like virus 2	GenBank	NC_035120.1	Viruses, unclassified viruses
Wuchang cockroach Virus 4	GenBank	KX883626.1	Viruses, unclassified RNA viruses
Wuhan horsefly Virus 3	GenBank	NC_033474.1	Viruses, unclassified RNA viruses
Wuhan insect virus 18	GenBank	KX883516.1	Viruses, unclassified RNA viruses
Zhejiang mosquito virus 3	GenBank	KX883461.1	Viruses, unclassified RNA viruses
Zhejiang mosquito virus 3	GenBank	KX883537.1	Viruses, unclassified RNA viruses
Zhejiang mosquito virus 3	GenBank	KX883538.1	Viruses, unclassified RNA viruses
Zhejiang mosquito virus 3	GenBank	MF176257.1	Viruses, unclassified RNA viruses
Zhejiang mosquito virus 3	GenBank	MF176278.1	Viruses, unclassified RNA viruses
Zhejiang mosquito virus 3	GenBank	MF176306.1	Viruses, unclassified RNA viruses
Zhejiang mosquito virus 3	GenBank	MF176344.1	Viruses, unclassified RNA viruses
Zhejiang mosquito virus 3	GenBank	MF176365.1	Viruses, unclassified RNA viruses
Zhejiang mosquito virus 3	GenBank	MF176385.1	Viruses, unclassified RNA viruses
Acartia tonsa	TSA	HAGX01098760.1	Eukaryota, Metazoa, Ecdysozoa, Arthropoda

Agarophyton chilense	TSA	GEZJ01006036.1	Eukaryota, Rhodophyta, Florideophyceae, Rhodymeniophycidae
Agarum clathratum	TSA	GEWO01002579.1	Eukaryota, Stramenopiles, PX clade, Phaeophyceae
Agarum clathratum	TSA	IABI01004424.1	Eukaryota, Stramenopiles, PX clade, Phaeophyceae
Agrostis stolonifera	TSA	GFQK01014503.1	Eukaryota, Viridiplantae, Streptophyta, Embryophyta
Bactrocera dorsalis	TSA	GEEA01024908.1	Eukaryota, Metazoa, Ecdysozoa, Arthropoda
Bactrocera dorsalis	TSA	GGBS01024784.1	Eukaryota, Metazoa, Ecdysozoa, Arthropoda
Brassica rapa subsp. chinensis	TSA	GFUS01019776.1	Eukaryota, Viridiplantae, Streptophyta, Embryophyta
Callosobruchus maculatus	TSA	GEUE01035234.1	Eukaryota, Metazoa, Ecdysozoa, Arthropoda
Callosobruchus maculatus	TSA	GEUE01057748.1	Eukaryota, Metazoa, Ecdysozoa, Arthropoda
Caridina multidentata	TSA	IABX01132835.1	Eukaryota, Metazoa, Ecdysozoa, Arthropoda
Ceratina chalybea	TSA	GBPU01010966.1	Eukaryota, Metazoa, Ecdysozoa, Arthropoda
Ceratitis capitata	TSA	GAMC01013668.1	Eukaryota, Metazoa, Ecdysozoa, Arthropoda
Colobanthus quitensis	TSA	GCIB01004342.1	Eukaryota, Viridiplantae, Streptophyta, Embryophyta
Colpodella angusta	TSA	GDKJ01026489.1	Eukaryota, Alveolata, Colpodellidae, Colpodella
Cronartium ribicola	TSA	GBSG01012161.1	Eukaryota, Fungi, Dikarya, Basidiomycota
Cronartium ribicola	TSA	GBSG01013692.1	Eukaryota, Fungi, Dikarya, Basidiomycota
Crossocerus quadrimaculatus	TSA	GBWH01015246.1	Eukaryota, Metazoa, Ecdysozoa, Arthropoda
Dendroctonus ponderosae	TSA	GDAR01017830.1	Eukaryota, Metazoa, Ecdysozoa, Arthropoda
Entomophthora muscae	TSA	GENB01008321.1	Eukaryota, Fungi, Zoopagomycota, Entomophthoromycotina
Entomophthora muscae	TSA	GENC01006608.1	Eukaryota, Fungi, Zoopagomycota, Entomophthoromycotina
Entomophthora muscae	TSA	GENC01041260.1	Eukaryota, Fungi, Zoopagomycota, Entomophthoromycotina
Entomophthora muscae	TSA	GEND01007370.1	Eukaryota, Fungi, Zoopagomycota, Entomophthoromycotina
Entomophthora muscae	TSA	GEND01011317.1	Eukaryota, Fungi, Zoopagomycota, Entomophthoromycotina
Eurypanopeus depressus	TSA	GFJG01044014.1	Eukaryota, Metazoa, Ecdysozoa, Arthropoda
Eurypanopeus depressus	TSA	GFJG01122116.1	Eukaryota, Metazoa, Ecdysozoa, Arthropoda
Fopius arisanus	TSA	GBYB01012090.1	Eukaryota, Metazoa, Ecdysozoa, Arthropoda
Gnathonemus petersii	TSA	GFIK01043228.1	Eukaryota, Metazoa, Chordata, Craniata
Helicoverpa assulta	TSA	GBTA01004534.1	Eukaryota, Metazoa, Ecdysozoa, Arthropoda
Heterosiphonia pulchra	TSA	GFLH01017038.1	Eukaryota, Rhodophyta, Florideophyceae, Rhodymeniophycidae
Hordeum vulgare	TSA	GGCO01034162.1	Eukaryota, Viridiplantae, Streptophyta, Embryophyta
Hordeum vulgare	TSA	GGCO01105932.1	Eukaryota, Viridiplantae, Streptophyta, Embryophyta
Humulus lupulus var. lupulus	TSA	LA534138.1	Eukaryota, Viridiplantae, Streptophyta, Embryophyta
Humulus lupulus var. lupulus	TSA	LA710658.1	Eukaryota, Viridiplantae, Streptophyta, Embryophyta
Idiosepius notoides	TSA	GFNE01046070.1	Eukaryota, Metazoa, Lophotrochozoa, Mollusca
insect metagenome	TSA	GCRV01019573.1	unclassified sequences, metagenomes, organismal metagenomes
Juncus effusus	TSA	GFBP01062652.1	Eukaryota, Viridiplantae, Streptophyta, Embryophyta

Lasioglossum xanthopus	TSA	GBPT01016202.1	Eukaryota, Metazoa, Ecdysozoa, Arthropoda
Lasioglossum xanthopus	TSA	GBPT01024369.1	Eukaryota, Metazoa, Ecdysozoa, Arthropoda
Lates calcarifer	TSA	GAMU01000156.1	Eukaryota, Metazoa, Chordata, Craniata
Leucoagaricus gongylophorus	TSA	HAAN01020927.1	Eukaryota, Fungi, Dikarya, Basidiomycota
Leucocoprinus sp. HH-2015a	TSA	GEHH01003501.1	Eukaryota, Fungi, Dikarya, Basidiomycota
Locusta migratoria	TSA	GBFE01004865.1	Eukaryota, Metazoa, Ecdysozoa, Arthropoda
Locusta migratoria manilensis	TSA	GBDZ01000351.1	Eukaryota, Metazoa, Ecdysozoa, Arthropoda
Loxostege sticticalis	TSA	GFCJ01029165.1	Eukaryota, Metazoa, Ecdysozoa, Arthropoda
Murgantia histrionica	TSA	GECQ01195387.1	Eukaryota, Metazoa, Ecdysozoa, Arthropoda
Myxobolus cerebralis	TSA	GBKL01051391.1	Eukaryota, Metazoa, Cnidaria, Myxozoa
Myxobolus cerebralis	TSA	GBKL01052498.1	Eukaryota, Metazoa, Cnidaria, Myxozoa
Nephila clavipes	TSA	GFKT011160020.1	Eukaryota, Metazoa, Ecdysozoa, Arthropoda
Nereocystis luetkeana	TSA	GEWH01000774.1	Eukaryota, Stramenopiles, PX clade, Phaeophyceae
Nereocystis luetkeana	TSA	GEWH01001802.1	Eukaryota, Stramenopiles, PX clade, Phaeophyceae
Nereocystis luetkeana	TSA	GEWH01003494.1	Eukaryota, Stramenopiles, PX clade, Phaeophyceae
Panax notoginseng	TSA	GFRX01320111.1	Eukaryota, Viridiplantae, Streptophyta, Embryophyta
Pergagrapta polita	TSA	GBWZ01004682.1	Eukaryota, Metazoa, Ecdysozoa, Arthropoda
Phakopsora pachyrhizi Thai1	TSA	GACM01002912.1	Eukaryota, Fungi, Dikarya, Basidiomycota
Pleurophycus gardneri	TSA	GEWL01001678.1	Eukaryota, Stramenopiles, PX clade, Phaeophyceae
Proasellus karamani	TSA	HAFC01092190.1	Eukaryota, Metazoa, Ecdysozoa, Arthropoda
Prosopis cineraria	TSA	GFOW01018115.1	Eukaryota, Viridiplantae, Streptophyta, Embryophyta
Puccinia striiformis f. sp. tritici	TSA	GAIR01012025.1	Eukaryota, Fungi, Dikarya, Basidiomycota
Puccinia striiformis f. sp. tritici	TSA	GAIR01012062.1	Eukaryota, Fungi, Dikarya, Basidiomycota
Puccinia striiformis f. sp. tritici	TSA	GAIS01005902.1	Eukaryota, Fungi, Dikarya, Basidiomycota
Pyropia haitanensis	TSA	GADD01004427.1	Eukaryota, Rhodophyta, Bangiophyceae, Bangiales
Pyropia haitanensis	TSA	GFOL01000028.1	Eukaryota, Rhodophyta, Bangiophyceae, Bangiales
Pyropia haitanensis	TSA	GFOL01000185.1	Eukaryota, Rhodophyta, Bangiophyceae, Bangiales
Pyropia haitanensis	TSA	GFOL01002916.1	Eukaryota, Rhodophyta, Bangiophyceae, Bangiales
Pyropia haitanensis	TSA	GFOL01002977.1	Eukaryota, Rhodophyta, Bangiophyceae, Bangiales
Rhipidia sejuga	TSA	GEMJ01010140.1	Eukaryota, Metazoa, Ecdysozoa, Arthropoda
Rhizopus oryzae	TSA	GDUK01007882.1	Eukaryota, Fungi, Fungi incertae sedis, Mucoromycotina
Rhizopus oryzae	TSA	GDUK01014407.1	Eukaryota, Fungi, Fungi incertae sedis, Mucoromycotina
Saccharum hybrid cultivar	TSA	GFHZ01053830.1	Eukaryota, Viridiplantae, Streptophyta, Embryophyta
Saccharum hybrid cultivar	TSA	GFLP01304447.1	Eukaryota, Viridiplantae, Streptophyta, Embryophyta
Saccharum hybrid cultivar	TSA	GFLP01591397.1	Eukaryota, Viridiplantae, Streptophyta, Embryophyta
Saccostrea glomerata	TSA	GGIC01436417.1	Eukaryota, Metazoa, Lophotrochozoa, Mollusca
Tomicus yunnanensis	TSA	GFJU01140648.1	Eukaryota, Metazoa, Ecdysozoa, Arthropoda

Tracheliastes polycolpus	TSA	GGQW01011558.1	Eukaryota, Metazoa, Ecdysozoa, Arthropoda
Uromyces appendiculatus	TSA	GACI01002802.1	Eukaryota, Fungi, Dikarya, Basidiomycota
Zostera noltei	TSA	HACV01002003.1	Eukaryota, Viridiplantae, Streptophyta, Embryophyta
Zostera noltei	TSA	HACV01003050.1	Eukaryota, Viridiplantae, Streptophyta, Embryophyta

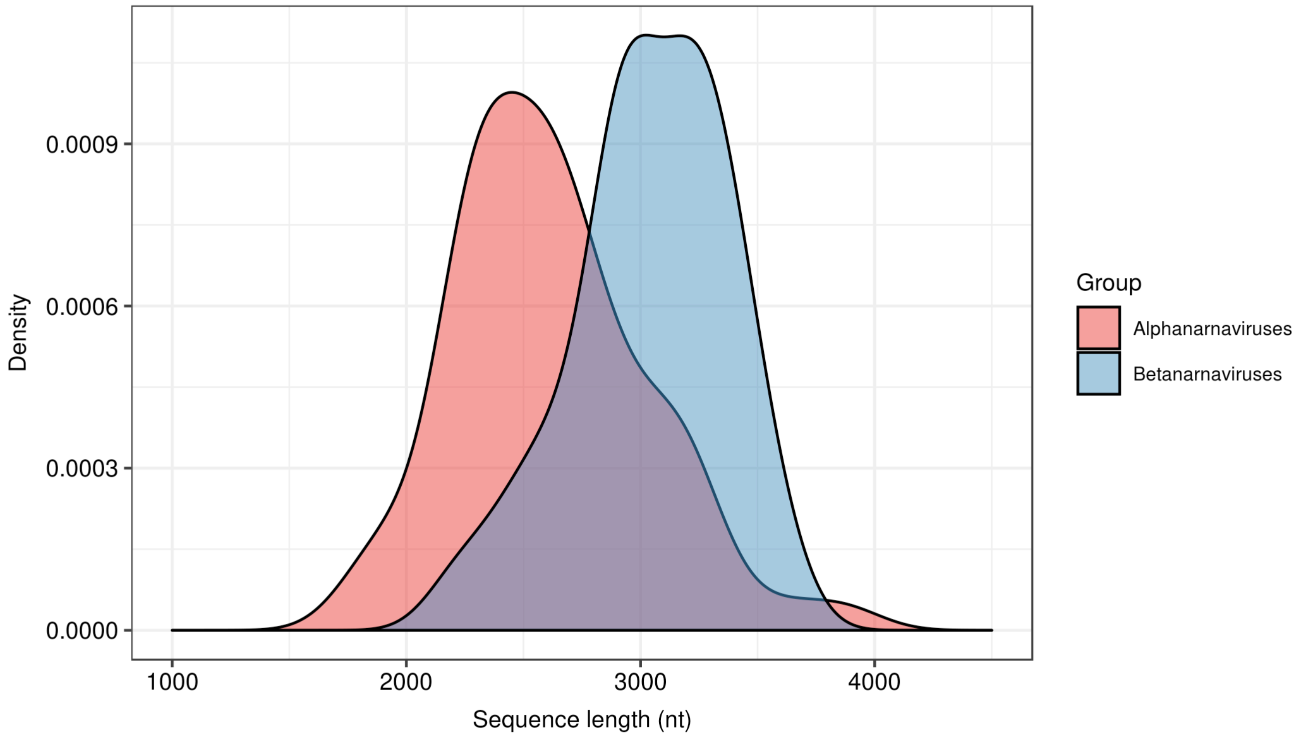

Figure S1. Gaussian kernal density estimates of the sequence length distributions for alphanarnaviruses (n = 64) and betanarnaviruses (n = 33) included in the analysis.

```
                    __ motif G __                 _________ motif F ________
GBSG01013692.1   SCFERSTTQGG-----VDGHLR // HCLRTPGYKCRVIGVPDALTFVEGTWVRWSSQLLPR   Cronr
MH213236.1       STFMTSMSEGG-----RLKEMM // IGVPEYGWKTRVLTLFPNYALTPGDLIR--QQLWPL   LhuNLV1
GFJU01140648.1   ATLNFHASQGG-----RMAELI // LVVPERGYKARVLVKFPASALLVGDIVR--RQLWPQ   Tomyu
KX883548.1       ASLNFKASEGG-----RLAELI // IAVSEKGYKARVLVEFPASTLLPGDIIR--RQLWPM   HNLV20
KX883542.1       ASQGATILEGG-----RMEELI // MAIAEKGYKARVLTKFPAAALVVGDVIR--RQMWAM   HNLV19
GGQW01011558.1   ASLEFTEKAGG-----RLADVM // VAVAEPGNKVRVVCKFRSVPLLISDIIR--RQLFPI   Trapo
GBFE01004865.1   ASRDYTIERGG-----RVQELL // MAAAEPGNKARVLCKFPAVALVPGDIIR--RQLWPI   Lmigr
KX883517.1       ASRDVPASAGG-----RLRDLV // LAIPEPGFKARVLCKFPATALLAGDIIR--RQLWPI   HNLV18
IABX01132835.1   ATLETTRAQGG-----FSEETR // IALAERGRKTRVVTKAPWEIVYLGHFLR--SWLLDG   Cmult
KX883500.1       ACKEVPRAKGG-----LGTRSV // MALPERGGKVRIVTKCPWALVYLGHFLR--VWLLEG   BNLV24
KX883605.1       ATFERTRTEGG-----FASQSV // LAIPERGLKARVVTKCPWALVYLGHFLR--SWLLQG   WNLV8
GFLP01591397.1   AVGERSKARGG-----YNAHIY // TGIGEQGDKCRIITVPPASLFAAGDVCR--SRIWPR   Sacch
GGCO01034162.1   ACYENPRGAGGFYAYIKKLGDK // AVIPERGYKNRVVTAPPASILSMGEVVR--SSIFPY   Horvu
GACI01002802.1   ACYEGPRSRGG-----YLGHIK // STIPERGYKNRIVTAPPSGVLSVGEVIR--SIIFPF   Uroma
GGCO01105932.1   SCFEGPRSKGG-----YFGYIR // GVVPERGYKNRIVTSPPSGVLSAGEVVR--HVLFPF   Horvu
GENC01041260.1   ---------------------- // VALRERGFKARIVTKSPVELVECGHLLR--SLVWPM   Enmus
LA534138.1       ATLDYSRRLGG-----MRSDLK // TTVKERGYKCRVVTKSPADVVEVGHLVR--SVVWPM   Humlu
GEUE01057748.1   ASVEFSRLKGG-----QTAELF // TVVPETGGKARVVTAGPADMVLAGNALR--RAVWPI   Calma
GBYB01012090.1   ASEGMSRAKGG-----QRAELE // TALPELGNKARVVTAPPAHWGIIGDAMR--KVLWPL   Fopar
KX883539.1       AAESVSRARGG-----QREELR // AVVPELGAKSRIVTAGEAHWVVIGDAIR--KCLWPA   HNLV21
GENC01006608.1   AAESVSRADGG-----QKADLL // SAIPEYGSKVRVVTACPGPFVVAGDACR--KIVWGL   Enmus
KF298275.1       AAEAFSAASGG-----QQAELR // TIVREQGMKARVVTACPAWAVVCGDACR--KTLWPL   ONLV1
KP642119.1       ASASVSAADGG-----QLAELR // TTVLELGMKARVVTKPPAWAVVAGDACR--QSVWPL   CNLV1
MF176385.1       ASATVSALKGG-----QLTELR // TVISELGMKARVVTKPPAWAVVAGDACR--KTVWPL   ZJMV3
KF298284.1       ASATVSAMSGG-----QLAELS // TVISELGMKARVVTKPPAWAVVAGNACR--KTVWPL   ONLV2
                                        //         * * *::         .    *     :
```

```
                   __ motif A__         _____motif B______       __motif C_               _motif D_  _motif E_
GBSG01013692.1   CSVDLSKATDGLSHD // VRGSPMGTPLSFIVLSWINSCA // -SIHGDDAVGT // LEEYKEFVRDIGATVNVSKTYISPTSFTMCERMY   Cronr
MH213236.1       VSCDLSNATDYVPHL // FRGTQMGTPLSFMTLCLLHRFA // -LIRGDDLIG- // PRIYMGMLEELGFKINKAKTFLSTIGGTFAERTF   LhuNLV1
GFJU01140648.1   VSADLSNATDYIPHL // KRGIHMGTPLSFMTLCLMHRYA // -IIRGDDLIA- // PDIYFSAMTSLGFKINKSKTIVSKNGGTFVERVF   Tomyu
KX883548.1       ISSDLSNATDYIPHL // QRGIHMGTPLSFMTLCLFHKFA // -LIRGDDLIG- // PARYCRTMEDLGFKINKSKTIISSKGGVFVERTF   HNLV20
KX883542.1       VSSDLSNATDYIPHI // ARGIHMGTPLSFLTLTLLHRFC // -IIRGDDLLG- // PEVYFNVMQQVGFSINRAKTIISRTGGTFAERTV   HNLV19
GGQW01011558.1   VSSDLSNATDYIPHH // QRGIQMGTPLSFMTLSLLHKFA // -LIRGDDLIG- // PRSYYSEMENLGLKINANKTLQSHRGGVFAEQTV   Trapo
GBFE01004865.1   VSSDLSNATDYIPHM // QRGIQMGTPLSFMTLSLLHKFA // -IIRGDDLLG- // PADYFRSMERVGFKINREKTIVSRVGGVFAEQTV   Lmigr
KX883517.1       VSSDLSNATDYIPHE // CRGIQMGTPLSFLTLSLLHKFC // -IIRGDDLLG- // PRQYLTVMEEIGFKINRDKTVISKDGGTFAEQTV   HNLV18
IABX01132835.1   LSADLTAASDLLPLD // ERGIMMGLPTTWCLLNLVQLFW // TAICGDDLVA- // IGRYEQVVKESGGMFSVGKHYRSGRYAVFTEQFY   Cmult
KX883500.1       VSADLTAASDLLPHD // QRGIMMGLPTTWTILSLVHLYW // TAICGDDLAA- // VQRYESIVKSCGGQFSAGKHYKSRTYLMFTEEAF   BNLV24
KX883605.1       LSADLTAATDRFPHD // ARGIMMGLPTTWCFLNLFNLFW // TVICGDDLAA- // CDQYERIAVACGASFSKGKHFRSARYLLFTEEPY   WNLV8
GFLP01591397.1   VSADLTKATDGFAHD // VRGILMGTPCSFIILSILNGWC // -VICGDDVAS- // VDNYDRRVTVIGSGLHKRKTFIGHKGLLFCELYV   Sacch
GGCO01034162.1   VSADLTKATDGFSHD // LRGSPMGTPCSFTLLCIVNSWA // --ICGDDMLA- // FSKYKVRVAAIGSGVHPLKTFISPYAGTFCENIY   Horvu
GACI01002802.1   YSADLTKATDGFSHE // RRGSPMGTPCSFTLLCILNLWS // --ICGDDMLA- // FAHYSRRISIIGSGVHVTKSYVSDIAGTFCENLY   Uroma
GGCO01105932.1   YSADLTKATDGFSHE // RRGSPMGTPCSFTLLCILNLWA // --ICGDDMLA- // FDSYSRRIAAIGSGVHPTKTFVSRSAGVFCENIY   Horvu
GENC01041260.1   ISADLTAATDGLYRW // KRGCLMGLPLSWFVLNVINIWA // -AICGDDLVG- // QRLYQRNIEVVGSGLSEGKHLESDRLAFFTEQAA   Enmus
LA534138.1       VSADLTKATDGFSRE // SRGCLMGLPLSWFVLNIINLWA // -AICGDDLAS- // HDGYERRISDVGSGLSDGKHLVSLHVLLFTEQMC   Humlu
GEUE01057748.1   YSGDLTAASDWLPRD // GAGALMGFPLTWLVLCAYNRAL // -VFRGDDMVS- // GARYEELVRLTGGQPNTAKSFRSVTGWVFTEATY   Calma
GBYB01012090.1   YSADLTAATDLMPEN // MRGCMMGLNLSWFLLNLYNLAI // -IVRGDDLAA- // ATAYEELIAATGGEANRAKSYRSHSAFVLAEKSF   Fopar
KX883539.1       YSADLTAATDLMPHD // HKGCMMGFPLSWYILNLYNLAC // -VVRGDDLCS- // ADRYERIIRATGGRANLSKSYRSRKGFILAERTF   HNLV21
GENC01006608.1   YSADLTAATDLMPFQ // QQGCMMGLPLSWTVLNVFNLAT // -VARGDDLAA- // ADRYEALISATGGRVNLSKSFRSSIGFVLAERTF   Enmus
KF298275.1       FSADLTAATDLMPFE // KQGCMMGLPLSWTILNLYNLAV // -IARGDDLVA- // ADRYTQLLRESGGEVNVLKSFRSSDSFVLAERTF   ONLV1
KP642119.1       FSADLTAATDDAP-- // VRGCMMGLPLSWTLLNLYNLAV // -IARGDDLVA- // ADRYEELIALTGGEANRLKSFRSATAFVLAERTF   CNLV1
MF176385.1       YSADLTAATDLMPFD // KRGCMMGLPVSWTVLNLYNLAM // -IARGDDLVA- // ATRYEDLIAATGGEANRLKSFRSADAFVLAERTF   ZJMV3
KF298284.1       YSADLTAATDLMPFD // SRGCMMGLPVSWTVLNLYNLAM // -VARGDDLVA- // ADRYENLIAATGGEANRLKSFRSTFAFVLAERTF   ONLV2
                  * **: *:*              *   **   ::  *   :          ***  .         *      *     *   .    : *
```

Figure S2. Alignment of RdRp protein-coding sequences of rORF-containing alphanarnaviruses. Known RdRp functional motifs are indicated and key functional residues previously described are highlighted. Key: Cronr = *Cronartium ribicola* TSA; LhuNLV1 = Linepithema humile narna-like virus 1; Tomyu = *Tomicus yunnanensis* TSA; HNLV = Hubei narna-like virus; Trapo = *Tracheliastes polycolpus* TSA; Lmigr = *Locusta migratoria* TSA; BNLV = Beihai narna-like virus; Cmult = *Caridina multidentata* TSA; WNLV = Wenling narna-like virus; Sacch = *Saccharum* hybrid TSA; Horvu = *Hordeum vulgare* TSA; Uroma = *Uromyces appendiculatus* TSA; Enmus = *Entomophthora muscae* TSA; Humlu = *Humulus lupulus* TSA; Calma = *Callosobruchus maculatus* TSA; Fopar = *Fopius arisanus* TSA; ONLV = Ochlerotatus-associated narna-like virus; CNLV = Culex-associated narna-like virus; ZJMV3 = Zhejiang mosquito virus 3.

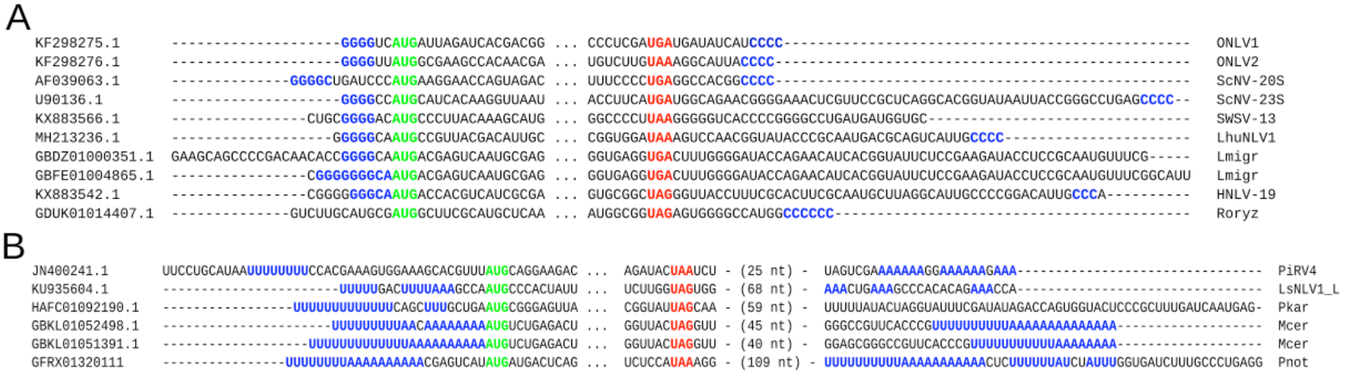

Figure S3. Alignments of **(A)** alphanarnaviral and **(B)** betanarnaviral genomic termini. The start and stop codons of the RdRp ORFs are shown in green and red, respectively. Complementary G/C- and A/U-rich regions are indicated in blue. Key: ONLV = Ochlerotatus-associated narna-like virus; ScNV-20S = Saccharomyces cerevisiae 20S narnavirus; ScNV-23S = Saccharomyces cerevisiae 23S narnavirus; SWSV-13 = Sanxia water strider virus strain 13; LhuNLV1 = Linepithema humile narna-like virus 1; Lmigr = *Locusta migratoria* TSA; HNLV-19 = Hubei narna-like virus 19; Roryz = *Rhizopus oryzae* TSA; PiRV4 = Phytophthora infestans RNA virus 4; LsNLV1_L = Leptomonas seymouri narna-like virus 1 segment L; Pkar = *Proasellus karamani* TSA; Mcer = *Myxobolus cerebralis* TSA; Pnot = *Panax notoginseng* TSA.

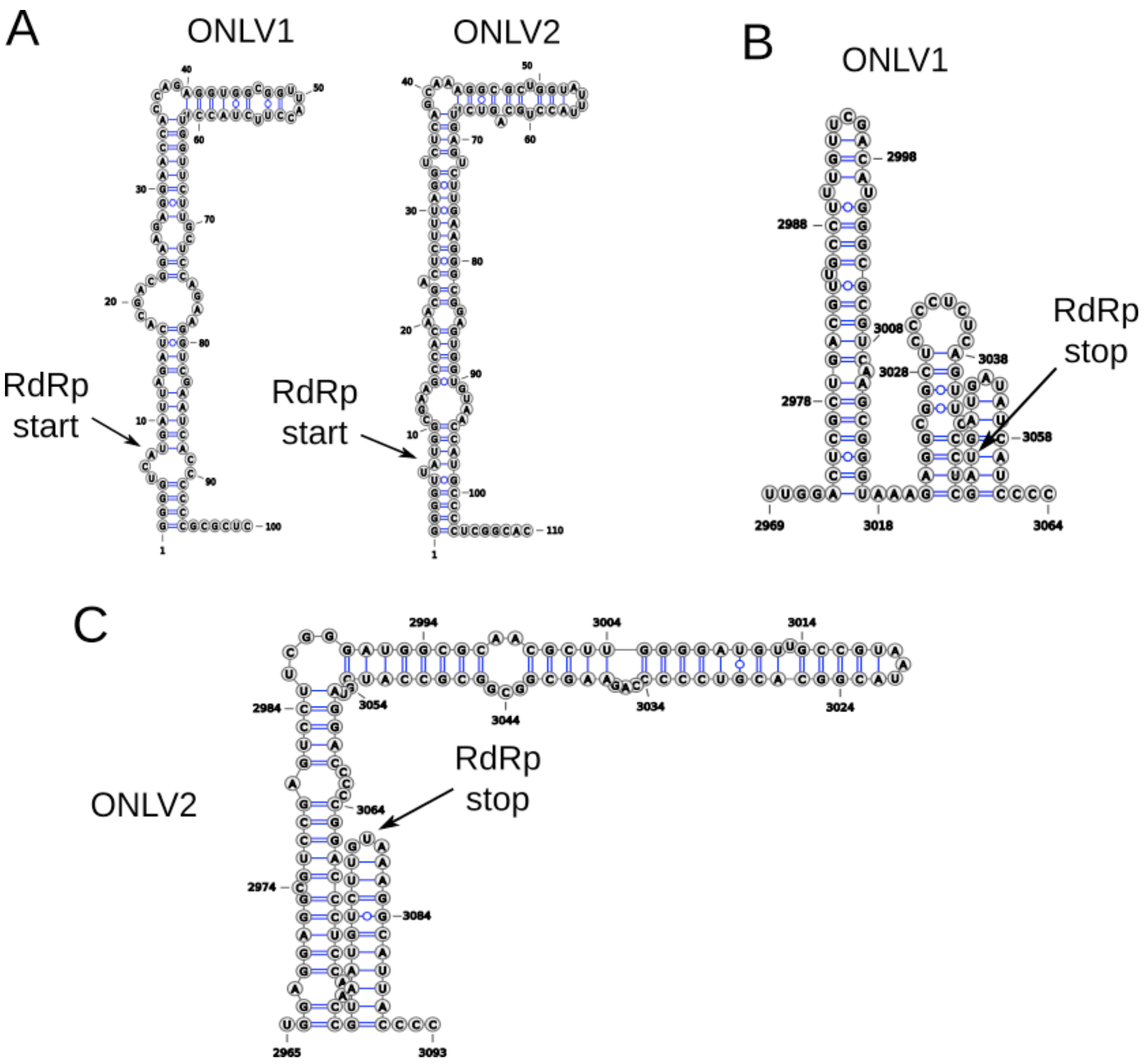

Figure S4. Predicted RNA secondary structures at the terminal regions of alphanarnaviral genomes. **(A)** 5′ termini of *Ochlerotatus*-associated narna-like virus (ONLV) 1 and ONLV2; **(B)** 3′ terminus of ONLV1; and **(C)** 3′ terminus of ONLV2.

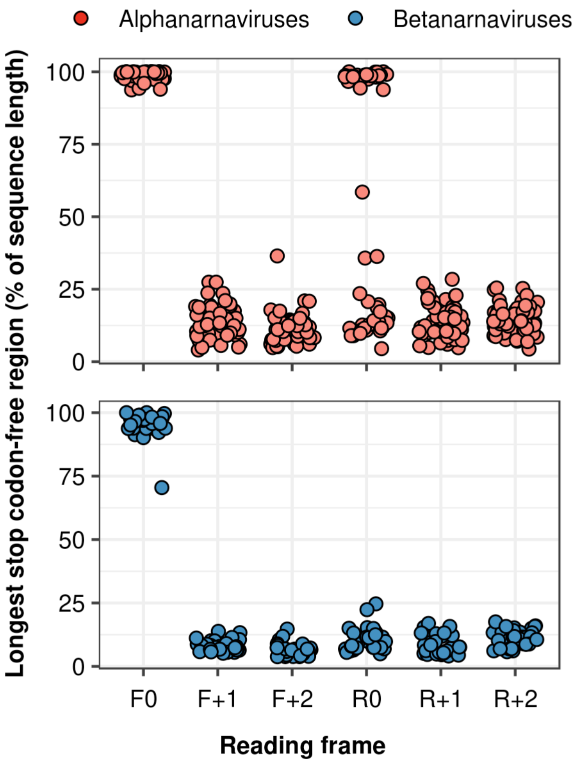

Figure S5. The longest stop codon-free regions in each of the three possible positive-strand and negative-strand reading frames (without the 5′-proximity restriction), for alphanarnaviruses (red) and betanarnaviruses (blue), as a percentage of the sequence length.

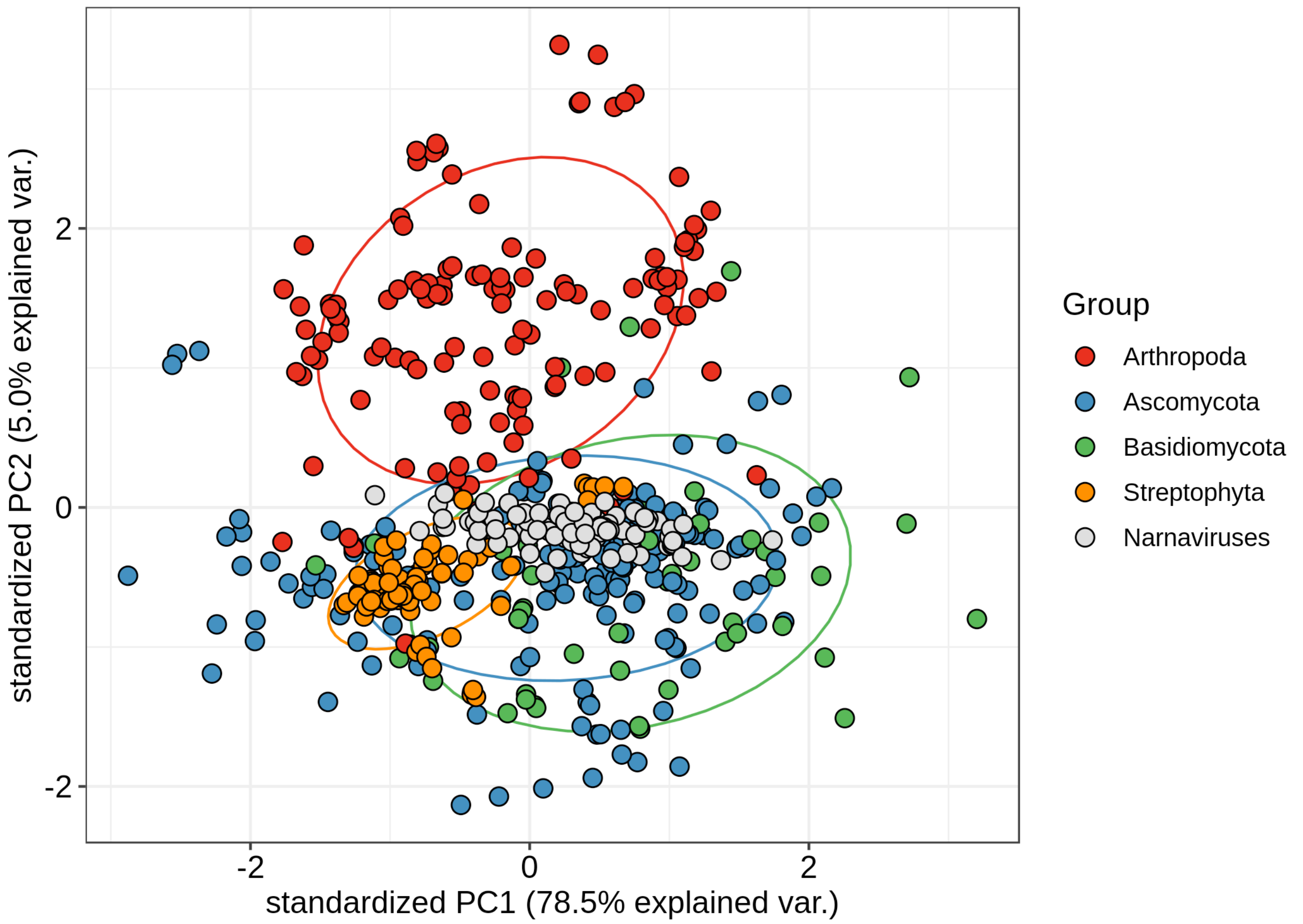

Figure S6. Principal component analysis of codon usage (per associated amino acid) in phyla that frequently co-occur with narnaviruses. Codon usage across NCBI RefSeq genomes was extracted from the latest release of the codon usage table database (CUTD). Ellipse contours are drawn for each group at a normal probability of 0.68.

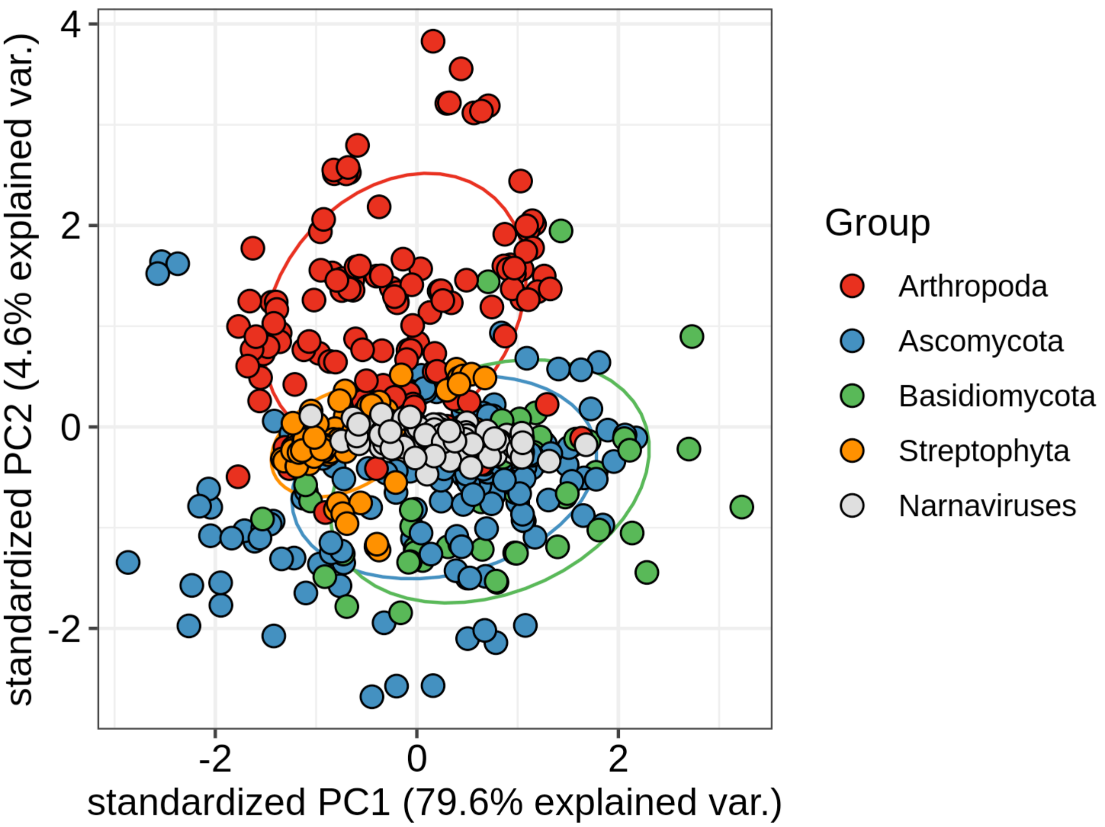

Figure S7. Principal component analysis of codon usage (per associated amino acid) in phyla that frequently co-occur with narnaviruses, excluding leucine and serine codons. Codon usage across NCBI RefSeq genomes was extracted from the latest release of the codon usage table database (CUTD). Ellipse contours are drawn for each group at a normal probability of 0.68.
